# Blockade of the C5a–C5aR axis alleviates lung damage in *hDPP4*-transgenic mice infected with MERS-CoV

**DOI:** 10.1038/s41426-018-0063-8

**Published:** 2018-04-24

**Authors:** Yuting Jiang, Guangyu Zhao, Nianping Song, Pei Li, Yuehong Chen, Yan Guo, Junfeng Li, Lanying Du, Shibo Jiang, Renfeng Guo, Shihui Sun, Yusen Zhou

**Affiliations:** 1 State Key Laboratory of Pathogen and Biosecurity, Beijing Institute of Microbiology and Epidemiology, Beijing, 100071 China; 2 Lindsley F. Kimball Research Institute, New York Blood Center, New York, NY 10065 USA; 3 Key Laboratory of Medical Molecular Virology of Ministries of Education and Health, Shanghai Medical College, Fudan University, Shanghai, 200032 China; 4 InflaRx GmbH, Jena, Germany

## Abstract

The pathogenesis of highly pathogenic Middle East respiratory syndrome coronavirus (MERS-CoV) remains poorly understood. In a previous study, we established an *hDPP4*-transgenic (*hDPP4*-Tg) mouse model in which MERS-CoV infection causes severe acute respiratory failure and high mortality accompanied by an elevated secretion of cytokines and chemokines. Since excessive complement activation is an important factor that contributes to acute lung injury after viral infection, in this study, we investigated the role of complement in MERS-CoV-induced lung damage. Our study showed that complement was excessively activated in MERS-CoV-infected *hDPP4*-Tg mice through observations of increased concentrations of the C5a and C5b-9 complement activation products in sera and lung tissues, respectively. Interestingly, blocking C5a production by targeting its receptor, C5aR, alleviated lung and spleen tissue damage and reduced inflammatory responses. More importantly, anti-C5aR antibody treatment led to decreased viral replication in lung tissues. Furthermore, compared with the sham treatment control, apoptosis of splenic cells was less pronounced in the splenic white pulp of treated mice, and greater number of proliferating splenic cells, particularly in the red pulp, was observed. These data indicate that (1) dysregulated host immune responses contribute to the severe outcome of MERS; (2) excessive complement activation, triggered by MERS-CoV infection, promote such dysregulation; and (3) blockade of the C5a–C5aR axis lead to the decreased tissue damage induced by MERS-CoV infection, as manifested by reduced apoptosis and T cell regeneration in the spleen. Therefore, the results of this study suggest a new strategy for clinical intervention and adjunctive treatment in MERS-CoV cases.

## Introduction

Middle East respiratory syndrome coronavirus (MERS-CoV) first emerged in November 2012 in Saudi Arabia^1^. As of February 9, 2018, MERS has been reported in 27 countries with 2143 laboratory-confirmed cases, including 750 deaths (~35% mortality rate). MERS-CoV is similar to severe acute respiratory syndrome coronavirus (SARS-CoV), which is in the same β-coronavirus genus as MERS-CoV and caused an outbreak during 2002–2003 with a 9.6% fatality rate^2^. MERS-CoV leads to progressive severe pneumonia in infected patients, with diffuse alveolar damage occurring during the acute phase. More severe infections are accompanied by considerable extrapulmonary organ dysfunction in the later phase^3–6^. SARS-CoV infections have been shown to lack type I interferon responses or cause lymphopenia with decreased numbers of CD4^+^ and CD8^+^ T cells during the acute phase^7^. However, the mechanism that contributes to severe clinical symptoms in MERS-CoV infections has still not been elucidated, owing to an absence of autopsy studies. The limited data from in vitro and ex vivo studies indicate that MERS-CoV induces a substantial cytopathic effect^8^ and dysregulation of host immune responses, similar to SARS-CoV^9, 10^.

Through comparative studies with SARS-CoV infection, immune-mediated pathogenesis has been proposed as a potential factor for severe outcome in MERS-CoV-infected patients^11, 12^. SARS patients showing hyper-immune activation, including aberrant expression of interferon (IFN)-stimulated genes and cytokine responses, are likely to succumb to SARS-CoV infection, the severity of which correlates with the amount of inflammatory cytokines present in the serum^13–15^. Clinical data on the pathogenesis of MERS-CoV infections are sparse. Josset et al.^6^ reported that MERS-CoV significantly downregulated the expression of genes involved in the antigen presentation pathway, which, in turn, could affect the development of adaptive immune responses. In addition, proinflammatory cytokines, such as interleukin-1β (IL-1β), IL-6, and IL-8, were markedly induced in Calu-3 cells after MERS-CoV infection^16^. Zhou et al.^10^ also speculated that cytokine storms could correlate with the severity of illness, since high levels of proinflammatory cytokines and chemokines were observed to be secreted by human macrophages upon MERS-CoV infection. Previously, we observed aberrant immune responses in MERS-CoV-infected *hDPP4*-Tg mice, such as elevated secretion of the cytokines IFN-γ-induced protein 10 (IP-10), IFN-γ, tumor necrosis factor-α (TNF-α), and IL-17, all of which are closely associated with virally mediated acute lung injury^14, 17–19^. Nevertheless, the role of MERS-CoV-induced excessive inflammatory responses as a factor contributing to the severity of disease outcome needs to be further studied.

The complement system plays an important role in host defense against microbial infection and maintains immune homeostasis. However, complement also contributes to the pathogenesis of many inflammatory and immunological diseases when excessively or inappropriately activated^20^. The biological effector functions of complement are mediated primarily through split products (i.e., C3a and C5a), which promote inflammation via direct and indirect mechanisms upon interaction with their respective receptors, C3a receptor and C5a receptor (C5aR)^21^. In addition to serving as a potent chemoattractant, C5a can also activate leukocytes, stimulate the release of granzymes, and stimulate phagocytosis and respiratory burst of mononuclear cells^22^. Furthermore, C5a induces mononuclear cells to express IL-1 and IL-8 in vitro and enhances IL-6 and TNF-α release in vivo^23^. Antibody (Ab) blockade of C5a or C5aR has been reported to abrogate excessive immune responses in a mouse model of *Plasmodium berghei* ANKA infection^24^. In our previous studies using H5N1-infected mouse models or an H7N9-infected non-human primate model, inhibition of over-activated complement system alleviated virus-induced acute lung injury^25, 26^.

High pathogenicity accompanied by increasingly severe dysregulation of immune responses in MERS-CoV-infected patients prompted us to explore the potential role of complement in the pathogenesis of MERS-CoV. Therefore, in this study, we demonstrate that MERS-CoV infection induced complement overactivation and led to severe lung damage, but that blockade of the interaction between C5a and C5aR alleviated tissue injury by re-establishing normal local and systemic immune responses, including reduced infiltration of tissue macrophages, inflammatory cytokine expression, and apoptosis of splenic cells and increased T cell regeneration. Therefore, it is suggested that complement inhibition may be a promising interventional strategy for treating MERS-CoV-infected patients.

## Materials and methods

### Ethics statement

All procedures involving animals were approved by the Laboratory Animal Center, State Key Laboratory of Pathogen and Biosecurity, Beijing Institute of Microbiology and Epidemiology IACUCs (Permit number: BIME 2017-0011). Animal studies were carried out in strict accordance with the recommendations in the Guide for the Care and Use of Laboratory Animals. All experimental operations on mice were performed under sodium pentobarbital anesthesia, and mice were euthanized by overdose inhalation of carbon dioxide.

### Mouse and virus strains

Six-week-old female *hDPP4*-Tg mice^27^ were maintained in a pathogen-free facility and housed in cages containing sterilized feed and drinking water. MERS-CoV (HCoV-EMC2012 strain) was propagated and titrated on Vero cells. Following intraperitoneal anesthetization with sodium pentobarbital (5 mg/kg of body weight), mice were treated intravenously (600 μg/kg, i.v.) with a monoclonal Ab (mAb) against mouse C5aR (Hycult Biotech, The Netherlands) or phosphate-buffered saline (PBS) as a sham treatment control. All mice were then intranasally inoculated with MERS-CoV (10^3.3^ 50% tissue culture infectious dose (TCID_50_)) in 20 μl Dulbecco’s modified Eagle’s medium. All infectious experiments related to MERS-CoV were performed in an approved biosafety level 3 facility.

### Histopathologic analysis of tissue damage

Lungs and spleens were collected and sampled in accordance with standard procedures. Sections 4 μm in thickness were stained with hematoxylin and eosin and examined by light microscopy. Lung tissue lesions were assessed according to the extent of denatured epithelial tissue, degeneration or necrosis of alveoli pneumocytes, infiltration of inflammatory cells, and expansion of parenchymal wall, hemorrhage, and interstitial edema^28^.

### Immunohistochemistry staining

Sections of paraffin-embedded spleen and lung tissues (4 μm in thickness) were prepared to detect antigen expression through immunohistochemistry (IHC) staining. Briefly, the retrieved sections were incubated overnight at 4 °C with the following antibodies: a mouse anti-C3 mAb (Hycult Biotech, The Netherlands), rabbit anti-C5b-9 (Calbiochem), and anti-C5aR (Santa Cruz Biotechnology, Paso Robles, CA, USA) polyclonal antibodies, rabbit anti-CD68 (Abcam, Cambridge, MA, USA) and anti-IFN-γRα (Santa Cruz Biotechnology) polyclonal antibodies, a rabbit cleaved caspase-3 Ab (Cell Signaling), a mouse anti-PCNA mAb (Santa Cruz Biotechnology), and a rabbit Novel coronavirus nucleoprotein/NP polyclonal Ab (Sino Biological Inc., Beijing, China). Biotinylated immunoglobulin G was then added, followed by an avidin–biotin–peroxidase conjugate (Beijing Zhongshan Biotechnology Co., Ltd.). Immunoreactivity was detected using 3,3' diamino benzidine (DAB) and by counterstaining with hematoxylin.

### Quantitative reverse transcription-PCR

To detect the expression of C5aR in lung tissue, lung samples were harvested and total RNA was extracted and purified using an RNeasy Extraction Kit (Qiagen, Germany). For each sample, 2 μg of total RNA was used as template for first-strand cDNA synthesis. The resulting cDNA was subjected to quantitative PCR using Power SYBR^®^ Green PCR Master Mix (Life Technologies, Carlsbad, CA, USA) to determine the relative abundance of C5aR in the tissues. The forward and reverse primers used for C5aR were previously described^26^. The relative amount of C5aR was determined by normalizing mRNA expression to that of the endogenous control gene *GAPDH*.

### Analysis of inflammatory cytokines and chemokines

Cytokines and chemokines in mouse sera were measured using a Milliplex Mouse Cytokine/Chemokine Magnetic Panel Kit (Merck Millipore, USA). A panel of inflammatory cytokines and chemokines (IL-1β, IL-6, TNF-α, IFN-γ, IL-10, IL-12, keratinocyte chemoattractant (KC), monocyte chemotactic protein-1 (MCP-1), and IP-10) were detected according to the manufacturer’s protocols.

### Detection of apoptosis in spleens

Sections of paraffin-embedded spleen (4 μm thickness) were prepared to assess splenic cell apoptosis using a TdT In Situ Apoptosis Detection Kit-DAB TUNEL (terminal deoxynucleotidyl transferase dUTP nick-end labeling)-based Apoptosis Detection Assay according to the manufacturer’s protocols (Roche, Germany). Briefly, after dewaxation and treatment with proteinase K, sections were labeled with labeling solution. After signal conversion using a Coverter-POD, the signal was detected and analyzed by light microscopy. The number of apoptotic cells in 10 random high-power fields (hpf) (×400 magnification) were calculated, and the results were expressed as TUNEL-positive cells/hpf.

### Viral titers in lung tissue

The lung tissues of infected mice were harvested aseptically at the indicated time points and homogenized in minimal essential medium (MEM) plus antibiotics to produce 10% (w/v) suspensions. Tissue homogenates were centrifuged and titered on monolayers of Vero cells. The cytopathic effects (CPEs) were observed daily via phase-contrast microscopy for 3 days. The viral titers were determined as TCID_50_ using a CPE-based assay and calculated using the Reed and Muench method. The viral titer was expressed as Log 10 TCID_50_/g of lung tissue.

### Statistical analysis

Statistical analyses were performed using the GraphPad Prism, version 5.01. Student’s *t* test was used to compare the treatment and sham groups to assess semi-quantitative histopathological damage, viral RNA copies, and titers in lungs, inflammatory cytokine/chemokine levels, and semi-quantitative splenocyte apoptosis and regeneration. To compare C5a and C5aR expression levels in sera and lung tissues, respectively, one-way analysis of variance with Dunnett’s post-test was used. The significance between survival curves was analyzed by Kaplan–Meier survival analysis with a log-rank test. *P* values lower than 0.05 were considered significant.

## Results

### MERS-CoV infection induces aberrant activation of complement, particularly the C5a–C5aR axis

**Fig. 1:**
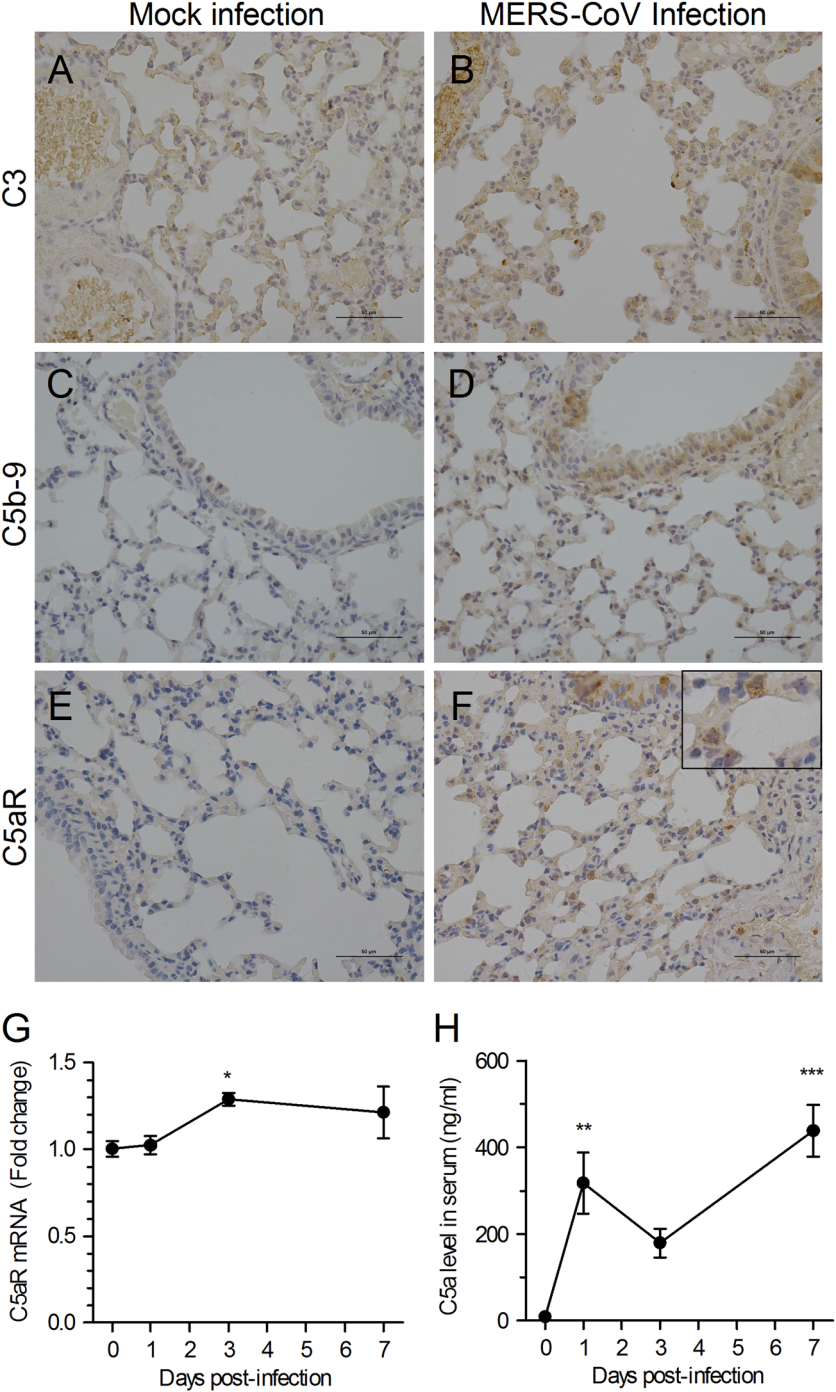
MERS-CoV infection induces excessive complement activation in *hDPP4*-transgenic mice. **a**–**f** Representative images of lung tissue sections from MERS-CoV-infected or Mock-infected *hDPP4*-transgenic mice by immunohistochemical staining for C3, C5b-9, and C5aR (*n* = 5 per group). **g** Transcriptional expression of C5aR in lung tissues at different time points after MERS-CoV infection (*n* = 3–5 per group). **h** Concentration of C5a in sera at different time points after virus infection was measured by a quantitative enzyme-linked immunosorbent assay (ELISA). Data are expressed as the means ± SEM (*n* = 5 per group). *P < 0.05, **P < 0.01, and ***P < 0.001 (one-way analysis of variance (ANOVA) with Dunnett’s post-test)

To determine whether local complement activation was related to lung damage after MERS-CoV infection, we examined complement activation in lung tissue. The results showed that deposition of C5b-9 increased after viral infection (Fig. 1a–d). The expression of C5aR in lungs was also increased, especially in the bronchus epithelial cells, pneumocytes, and inflammatory cells, as detected by immunohistochemistry staining (Fig. 1e, f). The relative expression of C5aR at the transcription level also confirmed the increased complement expression in lung tissue at day 3 post-infection (Fig. 1g). Furthermore, the presence of C5a in sera, as an indicator of complement activation, was detected by enzyme-linked immunosorbent assay. The results showed that after MERS-CoV infection, the C5a concentration increased at days 1 and 7 but decreased at day 3 post-infection (Fig. 1h). We speculate that MERS-CoV infection could promptly induce activation of both local and systemic complement activity. Subsequently, the virus-infected host may initiate a self-protection mechanism to suppress the over-activated complement response, resulting in the observed decrease in C5a production. However, continued viral replication may eventually overcome the host response, leading to a stronger complement activation and a rebound in C5a production. These findings suggest a close relationship between systemic inflammatory response and local tissue damage.

### Inhibition of complement activation with an anti-C5aR Ab reduces systemic and local inflammatory responses

**Fig. 2:**
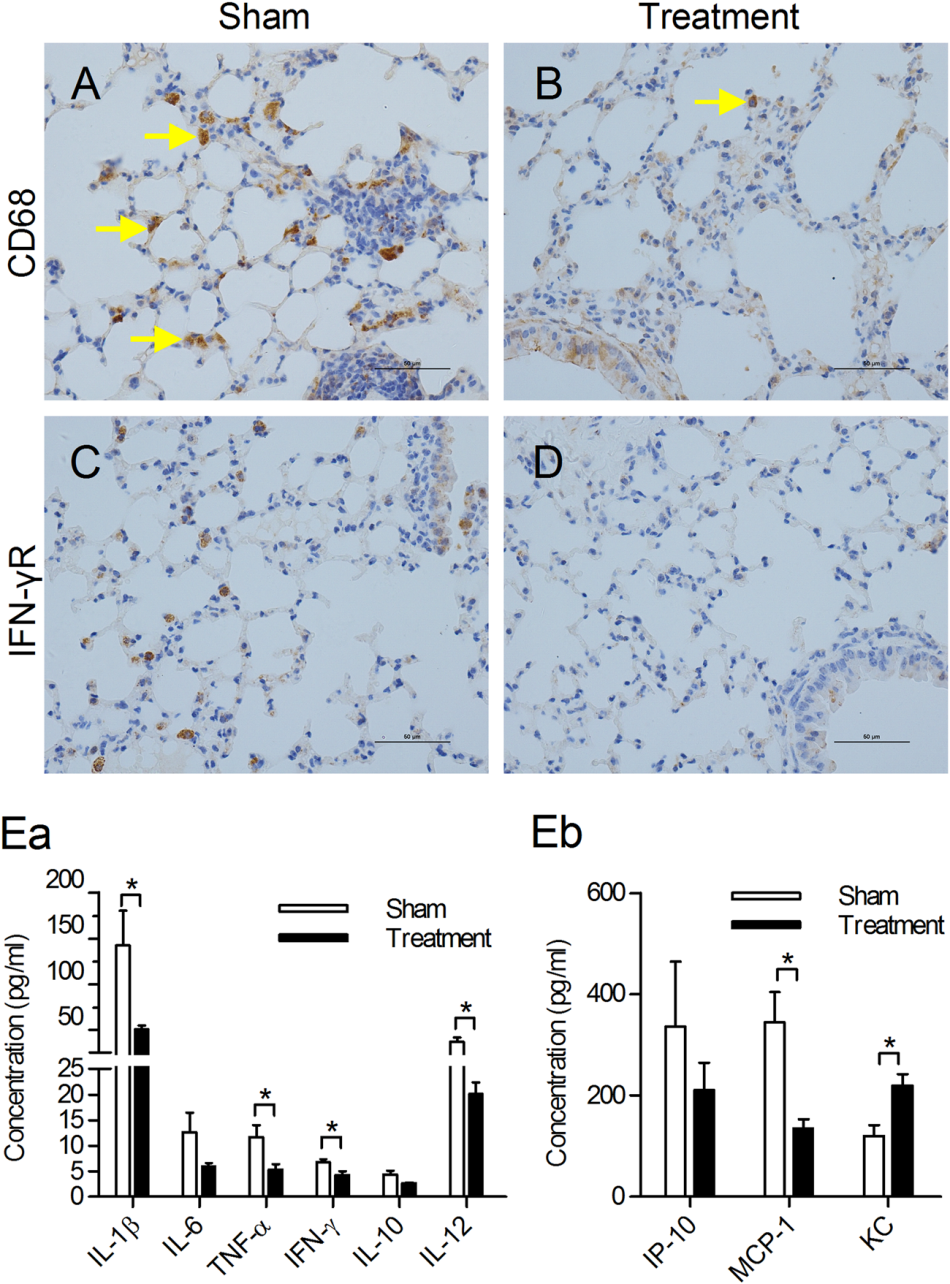
C5a–C5aR blockade reduced local and systemic inflammatory responses in *hDPP4*-transgenic mice. **a**–**d** Infiltration of macrophage (**a**–**b**) and the expression of IFN-γ receptor (**c**–**d**) were assessed by immunohistochemical staining in the lungs 3 days after challenge (CD68^+^ macrophages were indicated by arrows). **e** Sera from the two groups of mice were collected on day 3 and assayed for the levels of IL-1β, IL-6, TNF-α, IFN-γ, IL-10, IL-12 (**e**a), KC, MCP-1, and IP-10 (**e**b). The results are the mean ± SEM (*n* = 5). *P  < 0.05 (Student’s *t* test with Welch’s correction)

Although complement was promptly activated after viral infection, to confirm the efficacy of an anti-C5aR Ab treatment compared to a sham treatment control, we measured local and systemic inflammation in mouse lungs at day 3 post-infection. After treatment, macrophage infiltration was decreased in the lung tissues of mice (Fig. 2a, b). The expression of IFN-γ receptor, which is primarily expressed in inflammatory cells, was also decreased, as detected by immunohistochemistry (Fig. 2c, d). Furthermore, the concentration of proinflammatory cytokines, such as IL-1β, TNF-α, IFN-γ, and IL-12, decreased, and the levels of chemokines, such as MCP-1, significantly decreased in the anti-C5aR Ab treatment group. No significant difference in the concentrations of IL-6, IL-10, and IP-10 were detected between the treatment and control groups (Fig.  2ae, eb). Overall, the results suggested that anti-C5aR Ab treatment could decrease both local and systemic inflammation, especially the T-helper type 1 (Th1) immune response induced by MERS-CoV infection, which may also contribute to tissue damage after MERS-CoV infection.

### Inhibition of complement activation with an anti-C5aR Ab limits viral replication

**Fig. 3:**
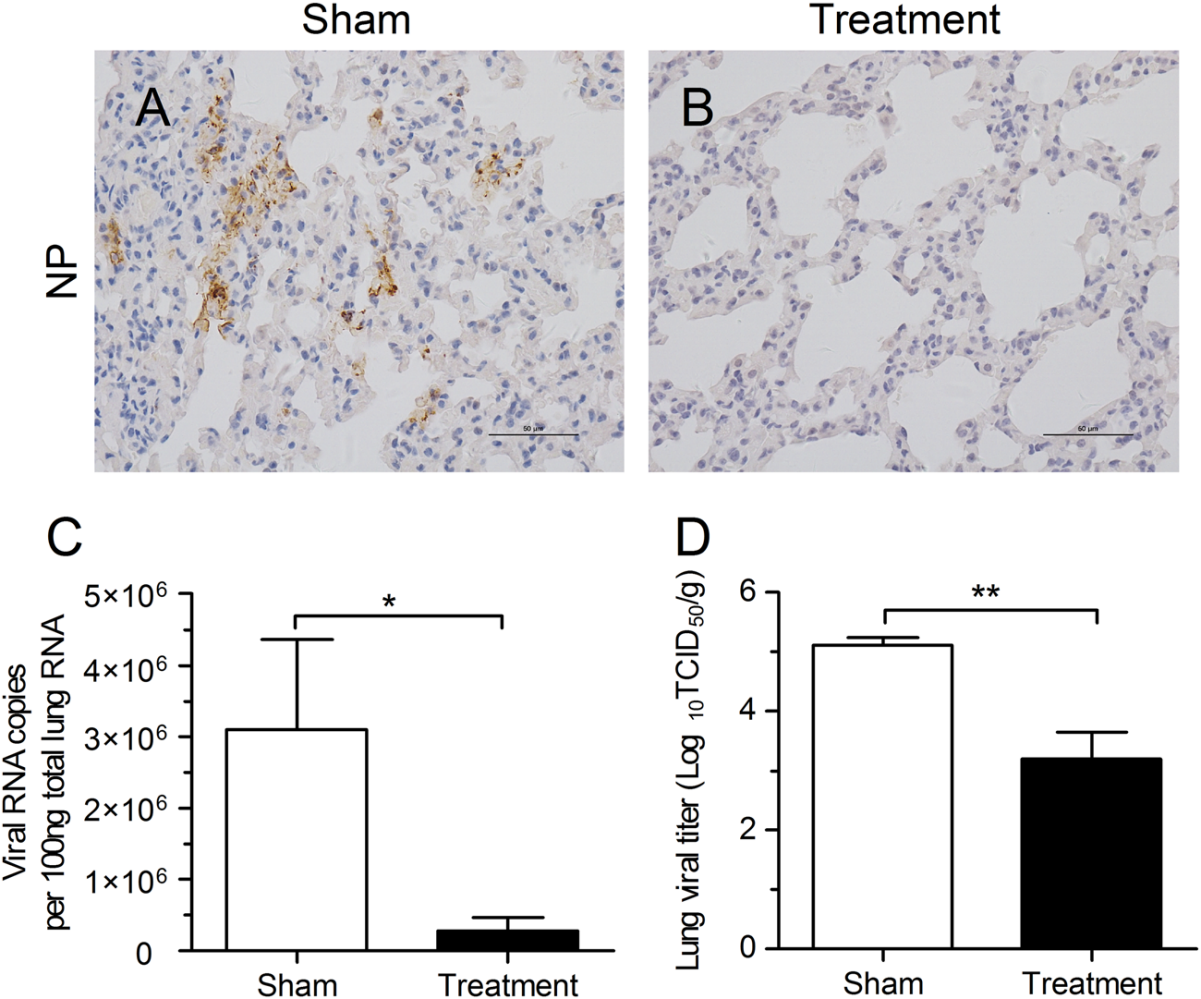
C5a–C5aR blockade limits viral replication in lung tissue. **a**–**b** Representative images of immunohistochemical staining of MERS-CoV antigen in lungs on day 7 after challenge in the sham treatment and anti-C5aR Ab treatment groups. **c** Viral RNA copies in lung tissues in the sham treatment and anti-C5aR Ab treatment groups. **d** Virus titer in lungs on day 7 after challenge in the sham treatment and anti-C5aR Ab treatment groups. Data are expressed as the means ± SEM (*n* = 5 per group). *P *<* 0.05, **P *<* 0.01 (Student’s *t*test with Welch’s correction)

To determine if complement activation influences viral replication, the viral titer in lung tissues, as well as antigen expression, was assessed. Compared to the PBS treatment group, the results showed that lung tissue exhibited less viral antigen expression (Fig. 3a, b) with lower viral titers and viral replication (Fig. 3c, d) in the anti-C5aR Ab treatment group. These results indicated that inhibition of complement activation could limit MERS-CoV replication in the lung.

### Inhibition of complement activation with an anti-C5aR Ab alleviates lung damage

**Fig. 4:**
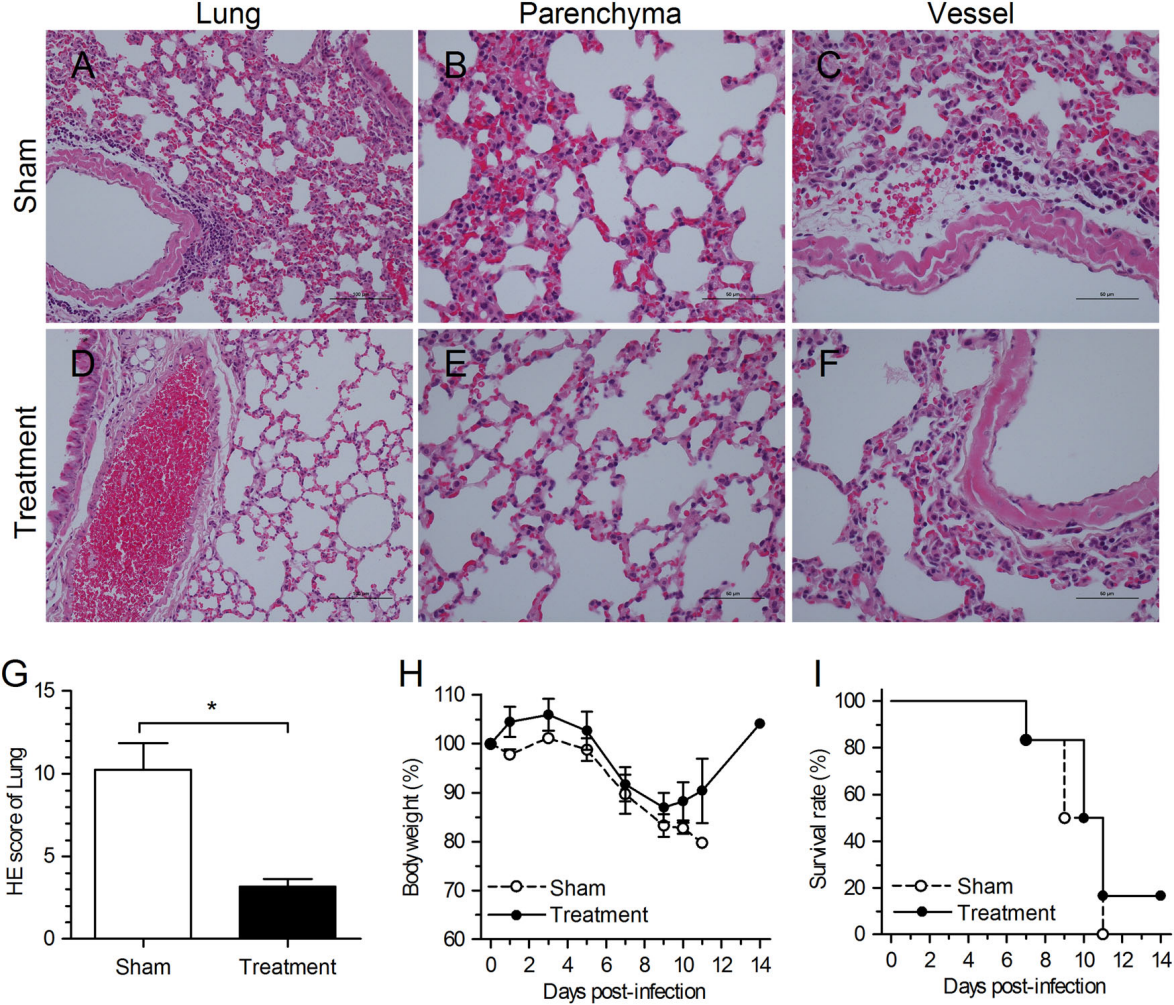
Alleviation of lung damage in *hDPP4*-transgenic mice after C5a–C5aR blockade. **a**–**f** Hematoxylin and eosin (H&E) staining of lung tissue sections obtained 7 days after anti-C5aR Ab treatment. Lung tissues in the sham treatment group presented mild to severe interstitial pneumonia, whereas those in the treatment group were less severe, with only mild focal thickening of alveolar septa. **g** Semi-quantitative histopathological analysis of H&E-stained lung sections 7 days after challenge. *P *<* 0.05 (Student’s *t*test with Welch’s correction). **h**–**i** Body weight and survival rate after challenge. An additional six mice in each group were weighed and monitored. The experiment was repeated once and data from one representative experiment of these two experiments is presented

The histopathology study at day 7 post-infection showed that MERS-CoV infection induced mild to severe interstitial pneumonia with diffused and thickened alveolar septa accompanied by infiltration of lymphocytes, macrophages, and neutrophils, denaturated and collapsed pneumocytes, and multifocal hemorrhage in the interstitial space of lungs (Fig. 4a–c). Therefore, to elucidate the role of excessive complement activation in infectious MERS-CoV-induced tissue damage, a mAb against the mouse C5a receptor was used to block the interaction of C5a with its receptor C5aR. After treatment, damage was lessened and only mild focal thickened alveolar septa was observed, especially around vessels, and no edema or hemorrhage in lungs was observed (Fig. 4d–f). A semi-quantitative analysis also confirmed the relatively less severe damage of lung tissues (Fig. 4g). In addition, after blockade, decreased weight loss was observed in the one mouse left alive compared with that observed in the sham treatment group (Fig. 4h, i), although the single mouse surviving treatment is not significant. The results further indicated that the blockade of the C5a–C5aR axis could alleviate lung damage in *hDPP4*-transgenic mice, although an antiviral agent inhibiting MERS-CoV infection may be required in combination with the anti-C5aR Ab to increase the survival rate of MERS-CoV-infected mice.

### Inhibition of complement activation with an anti-C5aR Ab decreases spleen damage by increasing splenic cell regeneration and decreasing splenic apoptosis

**Fig. 5:**
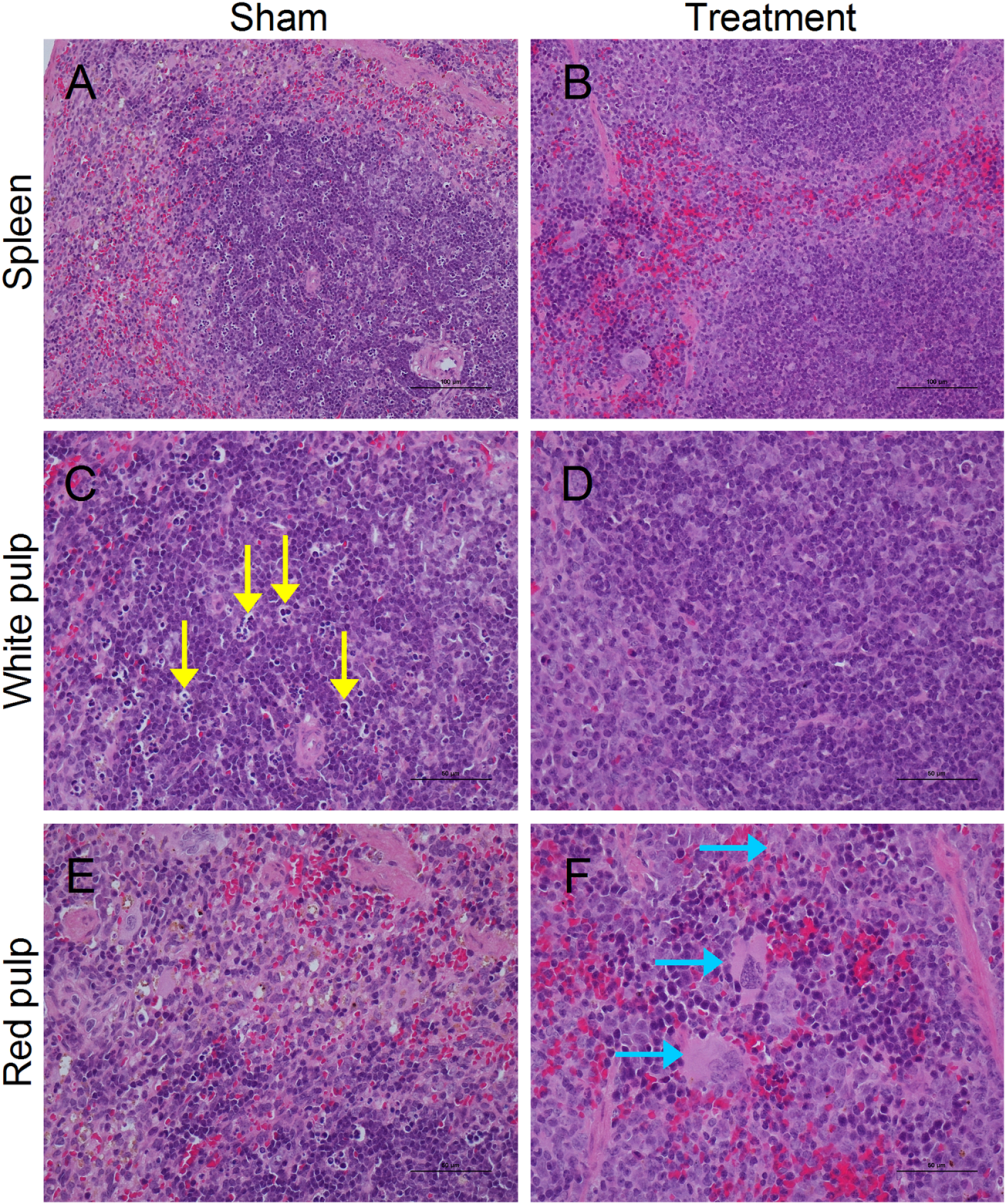
C5a–C5aR blockade decreased spleen damage in *hDPP4*-transgenic mice. **a**–**f** Representative images of spleen tissue sections with hematoxylin and eosin (H&E) staining on day 7 after viral challenge. Numerous splenic cells presented necrosis or apoptosis, especially in the white pulp (yellow arrow). Mice with the anti-C5aR Ab treatment had less spleen damage with less splenic necrosis and apoptosis, and increased numbers of macrophages were detected in red pulp (blue arrow) (*n* = 5 per group)

Clinically, MERS-CoV-infected patients have shown atypical pneumonia with dysregulated inflammatory responses. The spleen is an important peripheral lymphoid organ that is able to produce immune response immediately after viral infection. To test our hypothesis that blockade of the C5a–C5aR interaction may reduce spleen damage, spleen tissue was examined in MERS-CoV-infected *hDPP4*-transgenic mice with or without treatment with the anti-C5aR Ab. The results showed that a significant number of splenic cells presented necrosis or apoptosis, especially in the white pulp (yellow arrow), while numerous inflammatory cells infiltrated the red pulp (Fig. 5a, c, e). Interestingly, mice provided the anti-C5aR Ab treatment exhibited less spleen damage with less splenic cell necrosis or apoptosis and increased numbers of macrophages were detected in red pulp (blue arrow) (Fig. 5b, d, f). However, no MERS-CoV replication or viral antigen expression was observed in the spleens of mice in the treated and untreated groups (data not shown). Collectively, these findings suggest that the damages to the spleen tissue were not caused by MERS-CoV replication in the splenocytes but may result from the dysregulated immune and inflammatory responses caused by MERS-CoV infection in the lungs or other susceptible organs. Importantly, the anti-C5aR Ab treatment could significantly attenuate the spleen damage, suggesting that the dysregulated immune and inflammatory responses are likely closely associated with the complement system of the host.

**Fig. 6:**
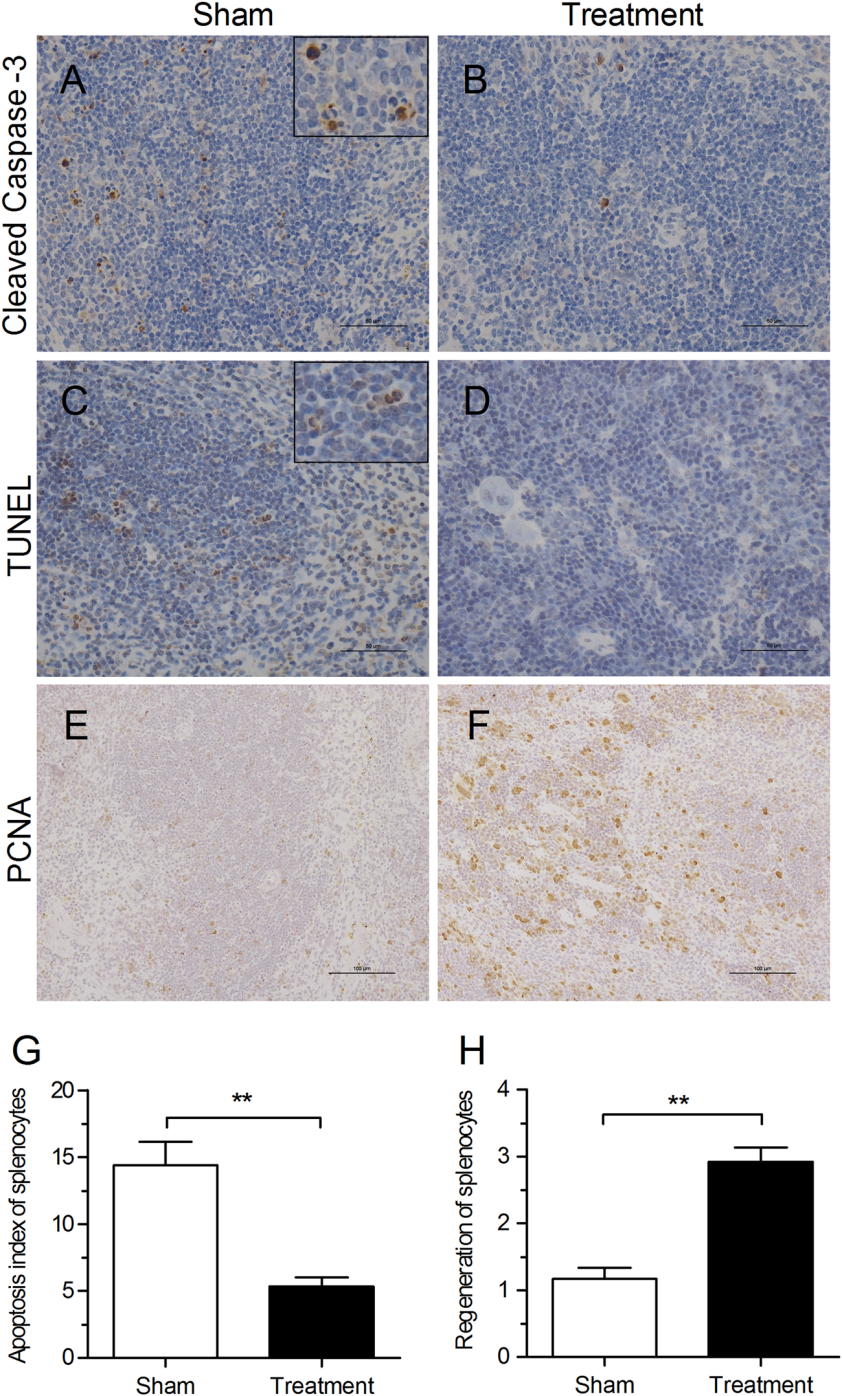
C5a–C5aR blockade in *hDPP4*-transgenic mice increases splenic cell regeneration and decreases splenic cell apoptosis. **a**, **b** Apoptosis of splenic cells was assessed by immunohistochemical staining of cleaved caspase-3 in spleen tissues 7 days after challenge. **c**, **d** Apoptosis of splenic cells was detected using a DAB TUNEL-based apoptosis detection assay in spleen tissue 7 days after challenge. **e**, **f** Representative images of regenerated splenic cells detected by IHC staining of PCNA 7 days after challenge. **g** Apoptosis index of splenocytes was assessed according to TUNEL-based apoptosis detection in spleen sections 7 days after challenge. **h** Semi-quantitative analysis of PCNA-positive cells in spleen sections 7 days after challenge (*n* = 5 per group)

Lymphopenia is a primary clinical aspect of severely ill patients^8^. After MERS-CoV infection in our *hDPP4-*transgenic mice, we observed increased apoptosis of splenic cells, as confirmed by the detection of apoptosis-positive cells by immunohistochemistry of cleaved caspase-3 and TUNEL methods (Fig. 6a, c). In comparison, less apoptosis of splenic cells was observed in spleens in the anti-C5aR Ab treatment group (Fig. 6b, d, g). More interestingly, increased splenic cell regeneration was clearly evident in the red pulp of mice after the anti-C5aR Ab treatment, which was confirmed by the detection of PCNA expression in spleens by IHC staining (Fig. 6e, f, h).

We next evaluated the effect of the anti-C5aR Ab treatment on viral titers and inflammation in the brain, including the olfactory bulb, cerebral cortex, and hippocampus, on day 7 post-infection. The results showed that there was less viral antigen and less activated microglia in the brains of the anti-C5aR Ab-treated mice compared with the untreated mice (data not shown). This result suggests that the anti-C5aR treatment could also suppress the inflammatory response and alleviate damage to brain tissues caused by MERS-CoV infection.

Collectively, these results suggested that the severe outcome of MERS-CoV infection is related to host lymphopenia, which may be closely related to the overactivation of the complement system. However, blockade of the C5a–C5aR axis could decrease damage to lung, spleen, and brain tissues caused by MERS-CoV infection.

## Discussion

According to a comparative study of SARS-CoV patients and the limited clinical data of MERS-CoV patients, immune-mediated pathogenesis was proposed as a potential factors in the severe outcome of MERS-CoV-infected patients. In this study, a human DPP4 transgenic (hDDP4-Tg) mouse model we previously developed was used to investigate whether MERS-CoV infection can cause aberrant systemic inflammatory responses^27^. One may question whether the human DPP4 protein expressed in the Tg mouse itself may cause dysregulated immune and inflammatory responses since hDPP4 can function as a signaling protein on the surface of T cells, as an enzyme cleaving chemokines or cytokines in the lungs and brain, and a as costimulatory molecule for inducing cell proliferation^29^. However, in our previous study, we observed that the hDPP4-Tg and wild-type C57BL/6 mice had very similar presentations of immune responses without MERS-CoV infection (data not shown). In addition, Tseng’s group did not observe dysregulated immune and inflammatory responses in the hDDP4-Tg mice before viral challenge^30^. Above all, these results suggested that the human DPP4 protein expressed in the TG mouse may not significantly influence immune and inflammatory responses in mice, possibly because of the differences in DPP4 between species. The results of this study confirm that aberrant systemic inflammatory responses, especially those mediated by the dysregulated complement system as induced by MERS-CoV infection, may contribute to the severe outcome of such infections. Furthermore, blocking the C5a–C5aR interaction and complement activation by blocking the C5a–C5aR axis could be a promising strategy for the adjunctive treatment of MERS-CoV infection.

Complement functions as an immune surveillance system that rapidly responds to viral infection and plays a pivotal role in inflammatory responses. Compared to patients with dengue fever (DF), severely ill patients with dengue hemorrhagic fever (DHF) and dengue shock syndrome have been shown to have higher levels of C3 cleavage products, specifically fD, which cleaves fB to yield the active (C3bBb) C3 convertase, and lower levels of fH, which inactivates the C3 convertase^31–33^. In addition, high levels of C3a, C5a, and sC5b-9 were observed to be present before plasma leakage occurred in DHF patients^34^. Moreover, expression of the complement inhibitor CD59-encoding gene was up-regulated more strongly in peripheral blood mononuclear cells from DF patients than in DHF patients^35^. These studies implicated the role of the complement system in the pathogenesis of severe forms of Dengue virus-associated diseases. In a Ross River virus (RRV)-induced complement-deficient mouse model, less severe tissue damage and disease symptoms following RRV infection also indicated an important role of complement in the RRV-induced pathogenesis^36^. In our previous study on highly pathogenic H5N1 influenza virus infection, dysregulated complement activation was observed to be accompanied by severe lung damage, which suggested that aberrant complement regulation may be an important causative factor after H5N1 virus infection^25^.

C5a is an important proinflammatory polypeptide that mediates strong proinflammatory and immune modulatory signals in many diseases. For example, C5a induces the expression of IL-1 and IL-8 in mononuclear cells in vitro, and it enhances the release of IL-6 and TNF-α in vivo^23^. In this study, aberrant complement activation was confirmed in the lung tissues of mice after MERS-CoV infection, which prompted us to investigate the contribution of complement in the outcome of MERS-CoV infection. As expected, by blocking the C5a–C5aR axis, lung injury was alleviated after MERS-CoV infection, with less tissue damage accompanied by decreased infiltration of lung tissue macrophages and decreased systemic inflammatory response, especially the Th1 immune response as detected by IL-12 levels in sera. Previous studies based on infected human macrophages and dendritic cells indicated that MERS-CoV significantly induced the expression of inflammatory and chemotactic cytokines, as well as chemokines such as IP-10, MCP-1, IL-8, and IL-12 in macrophages and dendritic cells^10, 37, 38^. The host immune response triggered by MERS-CoV infection is a double-edged sword. It may help to suppress viral infection, but can cause severe lung damage as shown in this study. Importantly, we determined that blockade of the C5a–C5aR interaction by an anti-C5aR Ab could significantly reduce alveolar macrophage infiltration and IFN-γ receptor expression in lung tissue, resulting in alleviation of tissue damage induced by the excessive immune response.

The spleen is an important secondary lymphoid organ with a well-organized structure, allowing the capture, processing, and presentation of antigens, ultimately leading to successful elimination of pathogens and the induction of adaptive immunity. Thus, the spleen is pivotal for maintaining blood homeostasis and producing immune responses to viral infection^39^. The spleen is divided into the white and red pulps, which are separated by the marginal zone, where vast splenic macrophage populations, such as marginal metallophilic macrophages, marginal zone macrophages, and red pulp macrophages are now considered to play important roles in the control of infection, as well as in the induction of innate and adaptive immunity^40^. For example, red pulp macrophages can prevent autoimmunity by producing anti-inflammatory cytokines, such as TGF-β and IL-10, and by inducing T-regulatory cells^41^. These cytokines may be important to curb an excessive immune response that could be deleterious to the host after pathogen clearance. In our study, increased numbers of macrophages in splenic red pulp and decreased IL-12 concentrations in sera were observed in the treatment group compared to the sham treatment group, indicating that the anti-C5aR Ab treatment may decrease the excessive complement immune response induced by MERS-CoV infection.

Clinically, lymphopenia occurs in many infectious diseases, such as influenza, HIV, hypotosis, and sepsis. Lymphopenia is also commonly observed in MERS patients. The complement inhibiting, decay-accelerating factor (CD55) has been shown to regulate CD8^+^ T cell immunity to virus infection^42^. Ward and colleagues^43^ and Ward^44^ demonstrated that C5aR plays a crucial role in the development of septic lymphopenia and that targeting C5a to prevent lymphopenia can be considered to restore normal immune responses in lieu of “after-the-fact” strategies. Chu et al^45^. demonstrated that MERS-CoV infection induced apoptosis of human primary T lymphocytes involved in the caspase-dependent apoptosis pathway. Our study confirmed that MERS-CoV contributed to apoptosis of splenic cells in vivo, accompanied by severe spleen damage and dysregulated systemic immune response, indicating that T cells play a role in controlling the pathogenesis of MERS-CoV infection. It is possible that regulation of the host immune response by complement improved the status of spleen tissue by decreasing splenic apoptosis and increasing the regeneration of splenic cells, indicating that induction of C5a production by MERS-CoV infection may contribute to the dysregulated immune responses, while blockade of the C5a–C5aR axis may improve outcomes by restoring normal immune responses.

In summary, our study demonstrated that MERS-CoV infection can result in a dysregulated host immune response, which then contributes to the severe outcomes observed after infection. However, blockade of the C5a–C5aR interaction could alleviate tissue damage induced by MERS-CoV infection through the regulation of the host immune response, especially the apoptosis and regeneration of T cells in spleens. Our study suggests a new effective clinical intervention and adjunctive treatment for MERS-CoV infection.
